# Viral-mediated gene delivery of TMBIM6 protects the neonatal brain via disruption of NPR-CYP complex coupled with upregulation of Nrf-2 post-HI

**DOI:** 10.1186/s12974-019-1559-4

**Published:** 2019-08-31

**Authors:** Desislava Doycheva, Ningbo Xu, Jiping Tang, John Zhang

**Affiliations:** 1 Department of Physiology and Pharmacology, Basic Sciences, School of Medicine, Loma Linda University, Loma Linda, CA 92354 USA; 2 Departments of Anesthesiology, Neurosurgery and Neurology, Loma Linda University School of Medicine, 11041 Campus Street, Risley Hall, Loma Linda, CA 92354 USA

**Keywords:** BI-1, NPR, CYP, ER stress, Hypoxia-ischemia

## Abstract

**Background:**

Oxidative stress, inflammation, and endoplasmic reticulum (ER) stress play a major role in the pathogenesis of neonatal hypoxic-ischemic (HI) injury. ER stress results in the accumulation of unfolded proteins that trigger the NADPH-P450 reductase (NPR) and the microsomal monooxygenase system which is composed of cytochrome P450 members (CYP) generating reactive oxygen species (ROS) as well as the release of inflammatory cytokines.

We explored the role of Bax Inhibitor-1 (BI-1) protein, encoded by the Transmembrane Bax inhibitor Motif Containing 6 (TMBIM6) gene, in protection from ER stress after HI brain injury. BI-1 may attenuate ER stress-induced ROS production and release of inflammatory mediators via (1) disruption of the NPR-CYP complex and (2) upregulation of Nrf-2, a redox-sensitive transcription factor, thus promoting an increase in anti-oxidant enzymes to inhibit ROS production. The main objective of our study is to evaluate BI-1’s inhibitory effects on ROS production and inflammation by overexpressing BI-1 in 10-day-old rat pups.

**Methods:**

Ten-day-old (P10) unsexed Sprague-Dawley rat pups underwent right common carotid artery ligation, followed by 1.5 h of hypoxia. To overexpress BI-1, rat pups were intracerebroventricularly (icv) injected at 48 h pre-HI with the human adenoviral vector-TMBIM6 (Ad-TMBIM6). BI-1 and Nrf-2 silencing were achieved by icv injection at 48 h pre-HI using siRNA to elucidate the potential mechanism. Percent infarcted area, immunofluorescent staining, DHE staining, western blot, and long-term neurobehavior assessments were performed.

**Results:**

Overexpression of BI-1 significantly reduced the percent infarcted area and improved long-term neurobehavioral outcomes. BI-1’s mediated protection was observed to be via inhibition of P4502E1, a major contributor to ROS generation and upregulation of pNrf-2 and HO-1, which correlated with a decrease in ROS and inflammatory markers. This effect was reversed when BI-1 or Nrf-2 were inhibited.

**Conclusions:**

Overexpression of BI-1 increased the production of antioxidant enzymes and attenuated inflammation by destabilizing the complex responsible for ROS production. BI-1’s multimodal role in inhibiting P4502E1, together with upregulating Nrf-2, makes it a promising therapeutic target.

**Electronic supplementary material:**

The online version of this article (10.1186/s12974-019-1559-4) contains supplementary material, which is available to authorized users.

## Background

Hypoxia-ischemia (HI) in the perinatal period is associated with long-term disabilities affecting 1–4 infants per 1000 births [2, 13, 40]. The most common cause of HI is intrauterine asphyxia, which may be brought on by placental artery clotting, abruption, or inflammatory processes [7]. In the event of prolonged abruption and extended period of HI, the neonate develops hypoxic-ischemic encephalopathy (HIE), causing irreversible brain injury [7].

A main contributing injury mechanism post-HI is the disruption of correct protein folding that subsequently triggers reactive oxygen species (ROS) accumulation, microglia activation, and inflammation [8, 14, 44]. Previous reports have shown inflammatory cytokine release to be significantly elevated in the full-term infant post-HIE, which is linked with further exacerbating the damage and results in poor neurodevelopmental outcome [7]. Therefore, inhibition of inflammation is an attractive target for new therapeutic strategies.

Correct folding of transmembrane proteins takes place in the endoplasmic reticulum (ER) which is composed of an elaborate system of chaperones and enzymes [37]. However, under stressful conditions, such as after HI injury, the number of unfolded proteins exceeds the capacity of the chaperones leading to the accumulation of unfolded proteins and ER stress [33]. The ER responds to this by activating the unfolded protein response (UPR), which triggers sensor proteins to recognize and ameliorate ER stress [33, 47, 50]. However, persisting ER stress leads to the over-activation of the UPR, a highly redox-dependent pathway that causes accumulation of ROS [15]. ROS are a natural byproduct of signaling pathways; however, during stress, ROS production is increased significantly, which causes oxidative stress and damage to the cell [15].

A major source of ROS production at the ER is from the microsomal monooxygenase (MMO) system which is composed of cytochrome P450 (CYP), NADPH-P450 reductase (NPR), and phospholipids [12, 15, 32]. Specifically, cytochrome P4502E1, a member of CYP, is associated with the production of large amounts of ROS, due to the leakage of electron transfer between P4502E1 and NPR, thus indicating an important role of these cytochromes during ER stress [15, 32].

Bax Inhibitor-1 (BI-1) is an evolutionary protein, encoded by the TMBIM6 gene, which mainly resides on the ER membrane. BI-1 is a member of the TMBIM family, associated with cytoprotection [45, 49], and has been suggested to regulate ER stress-induced ROS production and subsequent inflammatory cytokine production via two essential mechanisms [19]. First, BI-1 can directly inhibit ROS by disrupting the NPR-CYP complex, a major generator of ROS. BI-1 alters the electron flow from P4502E1 to NPR, thus destabilizing this complex and attenuating ROS accumulation [12, 15, 17]. The P4502E1 member was found to be significantly reduced and its upregulation attenuated after ER stress in BI-1 overexpressing cells [15]. Second, BI-1 can directly increase the production of anti-oxidant transcription factors such as nuclear factor erythroid 2-related factor 2 (Nrf-2) [17, 19]. Nrf-2 stimulates the production of antioxidant enzymes, heme oxygenase-1 (HO-1), which in turn blocks ROS, thereby attenuating inflammation and promoting cell survival. Cells that overexpressed BI-1 increased Nrf-2 and HO-1 expression while its protective effects were abrogated after inhibition of HO-1 [17].

The specific objective of this study was to establish that overexpression of BI-1 protein, with adenoviral-TMBIM6 vector, can attenuate the morphological and neurological consequences postneonatal HI via disruption of the NPR-CYP complex coupled with upregulation of Nrf-2 and HO-1, thus attenuating oxidative stress. HO-1 is a rate-limiting enzyme shown to be able to attenuate ROS accumulation. Increased levels of HO-1 may limit oxidative dysregulation which causes misfolding of ER proteins thereby decreasing the UPR. Our *main novelty* lies in the ability to upregulate BI-1 protein using an adenoviral vector carrying the TMBIM6 gene to induce overexpression of BI-1 in an in vivo HIE model and the mechanisms involved.

Given the lack of effective treatment options for neonatal HI injury, we hope to establish a novel role for BI-1 protein and ER stress in the pathophysiology of neonatal HI injury and help leverage this new understanding to design interventions that affect the outcome of neonatal HI patients. This work is essential and may help to change the clinical management for HI patients and provide a foundation for future research in other related diseases with similar pathologies.

## Materials and methods

### In vivo experiments

All protocols were approved by the Institutional Animal Care and Use Committee of Loma Linda University and with NIH guide for the Care and Use of Laboratory Animals. The animals were cared for and all studies conducted in accordance with the US Public Health Service’s Policy on Humane Care and Use of Laboratory Animals. Sprague-Dawley rat mothers, with litters of 10~12 pups, were purchased from Harlan Labs (Livermore, CA). All experiments adhere to the ARRIVE guidelines for reporting animal studies. A total of 166 unsexed rat pups weighing 15–20 g were used and kept in a temperature-controlled environment with regular light/dark cycle until they were ready for surgery at 10-day-old post-birth (P10). All rats were randomly assigned to the experimental groups which are shown in Additional file 1: Experimental Design.

#### Hypoxic-ischemic rat model

Hypoxic-ischemic (HI) injury was induced as previously described following the well-established Rice Vannucci model [41, 42]. Briefly, P10 unsexed rats were anesthetized with 3% isoflurane gas in the air and maintained at 2.5% isoflurane during surgery. Once the rat was fully anesthetized and unresponsive, the rat’s neck was prepared and draped using standard sterile techniques. A small midline neck incision on the anterior neck was made with a No. 11 blade surgical knife (approximately 3–5 mm in length). The right carotid artery was dissected, isolated from surrounding structures, and double ligated with 5-O surgical suture and cut between the ligations. The animal’s skin was sutured back to close the incision. Throughout the surgical and postoperative period, the temperature was controlled with heating blankets and incubators. Rats were then allowed to recover for 1 h on a heated blanket and then placed in a 2 L Erlenmeyer airtight flask, which was partially submerged in a 37 °C water bath to maintain a constant thermal environment. Rat pups were exposed to a gas mixture of 8% oxygen and 92% nitrogen for 90 min. Thereafter, the animals were returned to their mothers and monitored daily.

#### Time course evaluation of proteins

Time course expression of endogenous BI-1, P4502E1, NPR, pNrf-2, and HO-1 levels were measured at 6 h, 12 h, 24 h, and 72 h post-HI by western blot. Rats were randomly divided into groups, and the ipsilateral brain hemispheres were collected for western blotting. Sham animals underwent surgery; however, the artery was only isolated without being ligated or cut, and pups were euthanized at 72 h post-HI.

#### Viral administration

Human adenoviral-TMBIM6 vector (Ad-TMBIM6) (Vector Biolabs) was injected intracerebroventricularly (icv) at 2 μL containing 1.6 × 10^11^ PFU/mL or 1.7 × 10^11^ PFU/mL per injection [3] at 48 h pre-HI. For ICV administrations, rat pups were anesthetized with isoflurane and their heads placed in a stereotaxic head frame. Scalps were incised, and a burr hole (1 mm) drilled at 1.5 mm rostral and 1.5 mm lateral/right of bregma. A Hamilton syringe was inserted to a depth of 1.7 mm below the dura and a microinfusion pump infused 2 μL (0.1 μL/min) of the virus. The needle was then removed over 10 min after completion of the infusion, and the burr hole plugged with bone wax. As a control for the viral vector, rats were infused with Ad-GFP with similar concentrations. The control group is referred to as vehicle.

#### RNAi administration

Rats were anesthetized and placed on a stereotactic frame. Two microliters of BI-1 siRNA (300 pmol/μL, Sigma-Aldrich) or Nrf-2 siRNA or scramble siRNA (300 pmol/μL, Santa Cruz) were administered icv using a Hamilton syringe (10 μL, Hamilton Co) into the right lateral ventricle (1.5 mm posterior, 1.5 mm lateral to bregma and 1.7 mm down from the surface of the brain) at 48 h pre-HI at a rate of 0.3 μL/min [34]. The needle was left in place for 10 min after administration was completed and was then withdrawn slowly over 5 min to prevent backflow. The hole was sealed with bone wax, and the skin was sutured.

#### Infarct area measurements

TTC (2,3,5-triphenyltetrazolium chloride monohydrate) staining was performed at 72 h post-HI to determine the percentage of the infarcted area as previously described [25]. Animals were anesthetized, the brains were removed and sectioned into 2-mm slices. A total of five to six slices were cut per brain and were then immersed in 2% TTC solution until the brains turned pink-red (~ 5 min) at room temperature [20, 53]. Slices were then washed in PBS and fixed overnight in 10% formaldehyde solution and imaged. Image J software was used to calculate the percentage of the infarcted area.

The area of each slice was calculated using the formula: ((area of contralateral hemisphere − area of the non-infarcted ipsilateral hemisphere)/2 × (area of contralateral hemisphere))× 100 [5, 48]. The average of all five slices was taken as a representative of the percentage of infarcted areas for each animal. All experiments were performed in an unbiased blinded fashion.

#### Western blotting of brain tissue samples

Western blotting was performed at 6 h, 12 h, 24 h, and 72 h post-HI as previously described [10, 21, 34, 46]. Rats were anesthetized and transcardially perfused with 100 ml ice-cold PBS (pH 7.4). The brain was isolated and divided into contralateral and ipsilateral hemispheres, then immediately snap-frozen in liquid nitrogen, and stored in − 80 °C for further use. Western blot samples were prepared by homogenizing the ipsilateral hemispheres in RIPA lysis buffer (Santa Cruz Biotechnology) for 15 min followed by centrifugation, 14,000×*g* at 4 °C for 30 min. The supernatant was collected, and protein concentration was measured using a detergent compatibility assay (catalog number: 5000112, DC™ Protein Assay Kit II, Bio-Rad, USA).

Equal amounts of protein (30 μg, chosen as best loading amount based on previous studies) [26, 34, 38] were loaded on an 8~12% SDS-PAGE gel and electrophoresed and then transferred to a nitrocellulose membrane (0.2 μm). The membrane was blocked for 1 h and then incubated with primary antibodies overnight at 4 °C: BI-1 antibody (1:1000, Abcam), anti-P4502E1 (1:2500, Abcam), anti-NPR antibody (1:1000, Abcam), anti-pNrf-2/Nrf-2 antibody (1:1000, Abcam), anti-HO1 antibody (1:200, Santa Cruz Biotechnology), anti-ROMO1 antibody (1:1000, Abcam), anti-TNFα antibody (1:1000, Abcam), anti-IL-6 antibody (1:500, Abcam), anti-Il-1β antibody (1:500, Abcam), anti-HA tag antibody (1:4000, Abcam), and actin (1:4000, Santa Cruz Biotechnology). The following day, membranes were incubated at room temperature for 1 h with horseradish peroxidase-conjugated secondary antibodies (Santa Cruz Biotechnology) followed by chemiluminescence detection (ECL plus kit, Amersham Bioscience). The densitometric quantification of the bands was performed with Image J software, and results were expressed as relative density to actin.

#### Histology

Brains were post-fixed in formalin and then sectioned into 10-μm-thick coronal slices by cryostat (CM3050S; Leica Microsystems) for immunohistochemistry and dihydroethidium (DHE) staining.

##### Immunofluorescence staining

Rats were anesthetized at 72 h post-HI and transcardially perfused with PBS and 10% formalin. Brain sections were then post fixated with formalin overnight and dehydrated in 30% sucrose solution for 3–5 days after which brains were frozen in OCT. Immunofluorescence staining was performed as previously described [34]. The tissue mounted on the slides was washed with 0.1 M PBS three times for 5 min and then incubated in 0.3% Triton X-100 in 0.1 M PBS for 30 min at room temperature. The tissue was then washed once more by 0.1 M PBS for 5 min, three times, and primary antibodies were applied overnight at 4 °C: anti-BI-1 antibody (1:100, Abcam), anti-P4502E1 antibody (1:100, Abcam), anti-NPR (1:100, Abcam), anti-Nrf-2 (1:100, Abcam), anti-HA tag (1:50, Abcam), anti-Iba-1 (1:100, Abcam), anti-Il-1β (1:100, Abcam), and anti-MPO (1:100, Abcam). After washing with PBS, sections were incubated with appropriate secondary antibodies: anti-rabbit IgG-TR, anti-mouse IgG-FITC, anti-goat IgG-FITC, and anti-rabbit IgG-FITC (1:200) for 2 h at RT. Finally, slides were mounted using Vectashield Antifade with DAPI (catalog number: H-1200, Vector Laboratories Inc., USA) and visualized under a fluorescent microscope (Leica DMi8, Leica Microsystems), and Magna Fire SP system (Olympus) was used to analyze microphotographs. Quantification of Il-1β and MPO positive cells was manually counted in the peri-ischemic regions. A total of six sections/brain were averaged and expressed as the ratio of positive cells to total cells (percentage).

##### DHE staining

To evaluate ROS production, we stained brain slices with dihydroethidium (DHE) stain [31]. Slides were prepared as described above.

A stock solution of DHE was prepared by diluting a total of 25 g of DHE (Invitrogen) solid in 1 mL methanol. The working reagent was prepared by taking 25.3 μL of stock solution and diluting it in 1 mL methanol. After washing slides in PBS 1 × 10 min, a working reagent of DHE was applied to each brain section and slides were let stand in a dark room for 30 min. Slides were then washed in PBS 3 × 10 min, dried off, and then mounted using DAPI and coverslip. A Leica DMi8 fluorescent microscope was used to visualize the slides.

#### Behavioral analysis

Neurobehavioral tests were performed at 4 weeks using rotarod and water maze tests.

##### Rotarod

The rotarod assesses motor impairment. Animals were placed on a spinning bar to test their motor function. Rats underwent stationary, constant (5 rpm), and 5 rpm with 2 rpm acceleration tests. The latency to fall was recorded for each test [4, 11].

##### Water maze

Morris water maze test assesses learning, memory, and visual functions [4, 11]. The activity of animals’ swim paths was recorded and measured for quantification of distance, latency, and swimming speed for 5 days using a Video Tracking System SMART-2000 (San Diego Instruments Inc., CA).

### In vitro experiments

Rat microglial immortalized cell line, HAPI (Millipore Sigma) [54], was used at passage six through nine. Cells were cultured in FK12 media enriched with 15% horse serum, 2.5% FBS and 5 mL of penicillin was added. Cells were then placed in an incubator at 37 °C receiving 5% CO_2_ and 95% oxygen and allowed to grow till they reached 70% confluency with replacing media every 3 days. Cells were passaged before use for experiments. For Western blot, cells were plated at a density of 200,000 cells/well in a six-well plate.

Primary microglial cells were prepared from newborn rats at postnatal day 1 according to protocol and a previous study [22, 52]. Briefly, brain cortices were collected and kept in HBSS solution. Once dissected, tissue was placed in trypsin-EDTA for 15 min to digest it. F12 media with 15% FBS was used to neutralize the trypsin and stop the enzymatic reaction. The cell suspension was then strained through a 200-μm filter and plated on poly-ornithine-coated flasks and placed in an incubator at 37 °C and 5% CO_2_ and 95% oxygen. Cells were allowed to grow and attach with replacing media every 3 days. Primary microglial cells were harvested via mechanical agitation for 2 h at 10 days post initial plating. The isolated cells were then plated at the desired density in six-well plates coated with poly-ornithine for western blotting and immunofluorescence staining. For western blot, cells were plated at a density of 200,000 cells/well in a six-well plate. For immunofluorescence, cells were plated at a density of 2.5 × 10^4^ cells/18-mm coverlid.

#### OGD

Cells were grown in full growth media which was replaced with glucose-deprived media prior to being placed in a chamber. Cells were placed in a hypoxic chamber and flushed with 1% oxygen for 3 h. Exposure of cells to 3 h of OGD was chosen from our preliminary results (data not shown). Media was removed, and complete growth media was re-introduced to the cells. Cells were left in an incubator to recover for 18 h after which were prepared for cell death assay and western blotting [1, 36].

#### Cell death assay

To determine the percentage of viable cells, we used trypan exclusion as previously described [36]. Briefly, cells were scraped from the plates, centrifuged for 5 min, and then re-suspended in 10 mL complete growth media. Equal volumes of cell suspension were added to trypan blue; cells were then vortexed and let stand for 3 min. Ten microliters from the mixture was placed on cell counter slides, and slides were read using an automated cell counter using an average of six counts [35].

#### Western blotting for cells

Cells were collected 18 h after OGD and stored for western blotting as previously described [6, 36]. Briefly, cells were scraped, centrifuged, and then re-suspended in 1 mL PBS, after which they were centrifuged one more time at 14,000 rpm for 5 min. The remaining supernatant was removed after centrifugation, leaving only the pellet. RIPA lysis buffer was added with protease inhibitor cocktail and pipetted thoroughly until the pellet was fully dissolved. This suspension was then left on ice for 20 min following which it was centrifuged for 30 min. The supernatant was harvested while the pellet was discarded. Protein concentration was measured using Bradford assay, and SD-PAGE electrophoresis was performed as previously described [6]. Primary antibodies, BI-1 antibody (1:200, Abcam), anti-P4502E1 antibody (1:1000, Abcam), anti-Nrf-2 antibody (1:1000, Abcam), anti-TNFα antibody (1:1000, Abcam), anti-IL-6 antibody (1:500, Abcam), anti-Il-1β antibody (1:500, Abcam), and actin (1:4000, Santa Cruz) were applied to the membrane and left overnight.

#### Calculating MOI

The Multiplicity of Infection (MOI) was calculated to determine the total amount of infectious particles needed to infect one cell. MOI was calculated as follows: no. cells × desired MOI = total PFU (or plaque-forming units) needed; (total PFU needed)/(PFU/mL) = total milliliters of virus needed to reach your desired dose. A 100 MOI of Ad-TMBIM6 virus was chosen as the optimal amount needed to infect cells and provide protection from our preliminary results (data not shown).

#### siRNA transfection

Cells were allowed to differentiate and reach 80% confluency in poly-D-lysine-coated six-well plates prior to transfection. Both BI-1 and Nrf-2 siRNAs were prepared according to the manufacturer’s protocol (Sigma-Aldrich); a stock solution of 10 μM siRNA was prepared. From the siRNA stock, 4 μL was taken and mixed with 125 μL Opti-MEM. In a separate tube, 7 μL lipofectamine 3000 was mixed with 125 μL Opti-MEM. The solution from both tubes was combined and mixed well and left to sit at room temperature for 15–45 min. Growth media was removed from cells and replaced with 250 μL/well siRNA transfection solution as prepared above. An additional 1 mL Opti-MEM was added to each well and cells were then placed in an incubator for 5–7 h, following which media was removed and replaced with normal growth media.

### Statistical analysis

Statistical analysis was performed using one-way analysis of variances (ANOVA), followed by Tukey’s test using Graph Pad Prism (Graph Pad Software, San Diego). All values were presented as mean ± standard deviation (SD), with a *p* value less than 5% considered significant. Sample sizes were determined by the power of 0.8, *α* = 0.05, and a 20% standard deviation from the preliminary results. The estimated sample size was 6–8/group for in vivo studies and 4/group for cell culture studies. All rats were randomly assigned to each group, and all the investigators were blinded. Please refer to Additional files 4, 5, 6, 7, 8, 9, and 10 for detailed statistical analysis.

## Results

### Time course expression of endogenous proteins post-HI

**Fig. 1:**
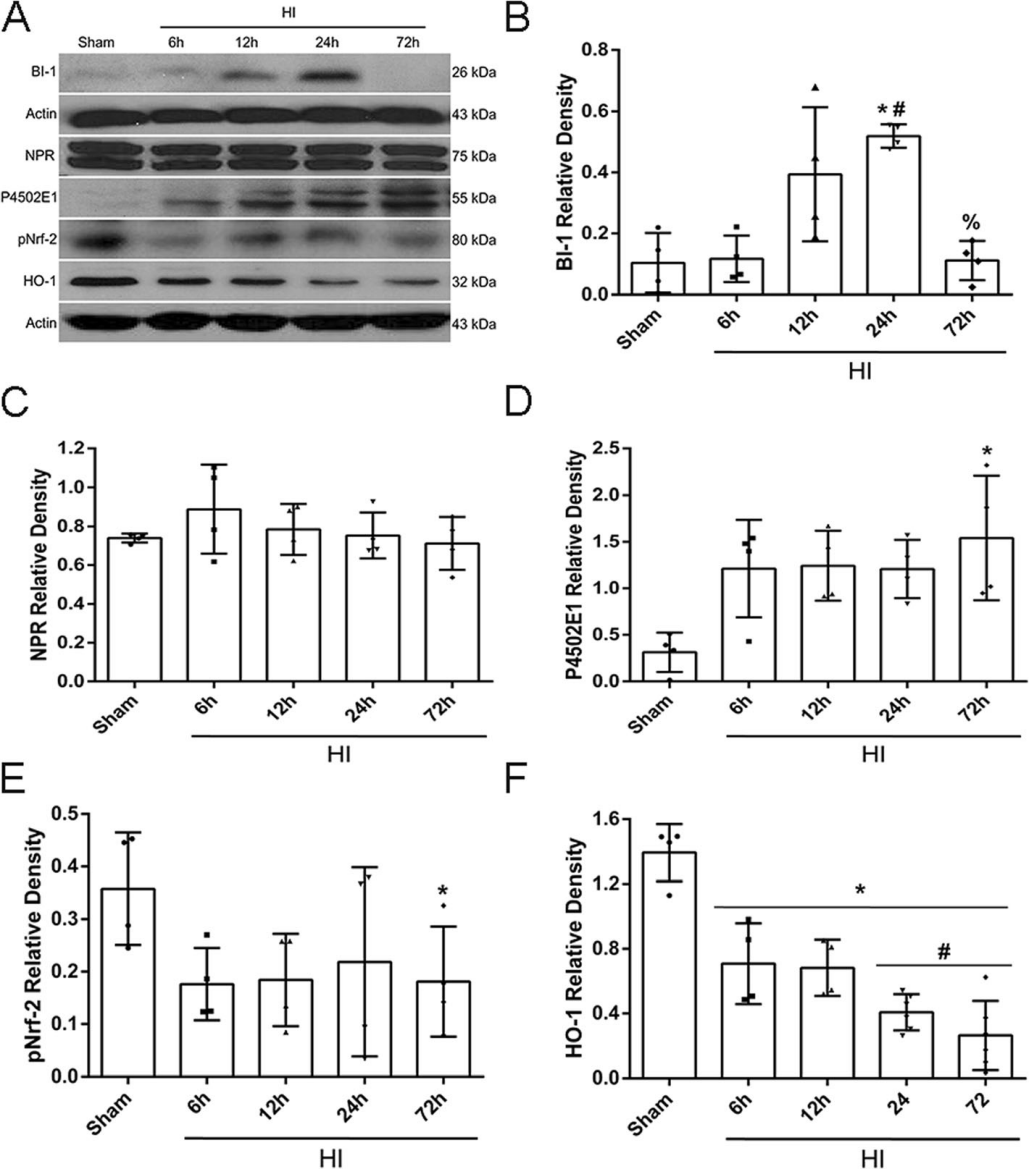
Endogenous expression profiles of BI-1, NPR, P4502E1, Nrf-2, and HO-1 post-HI. Representative western blot bands of the temporal expression of endogenous proteins in the ipsilateral hemisphere post-HI (**a**). Quantification data of western blot bands for BI-1 (**b**), NPR (**c**), P4502E1 (**d**), Nrf-2 (**e**), and HO-1 (**f**) (data expressed as mean ± SD; an asterisk indicates *p* < 0.05 vs sham; number sign *p <* 0.05 vs 6 h, @ *p*< 0.05 vs 24 h; *n* = 4, using one-way ANOVA followed by Tukey’s multiple-comparison post hoc analysis)

Endogenous expression of BI-1, NPR, P4502E1, pNrf-2, and HO-1 were measured at 6 h, 12 h, 24 h, and 72 h in the ipsilateral cerebral hemispheres post-HI. Results showed that BI-1 levels significantly increased at 24 h post-HI and returned to sham levels at 72 h post-HI (*p* < 0.05, Fig. 1a, b). There were no significant changes for NPR expression levels (Fig. 1b). P4502E1 significantly increased in a time-dependent manner, peaking at 72 h post-HI (*p* < 0.05, Fig. 1c). pNrf-2 showed a tendency to decrease in a time-dependent manner from 6 h to 72 h post-HI, and HO-1 levels significantly decreased from 6 h to 72 h, reaching significantly lower expression levels at 72 h post-HI compared to sham (*p* < 0.05, Fig. 1d, e). Please refer to Additional file 4 for detailed statistical analysis.

### Low dose Ad-TMBIM6 vector reduced infarct area at 72 h post-HI

**Fig. 2:**
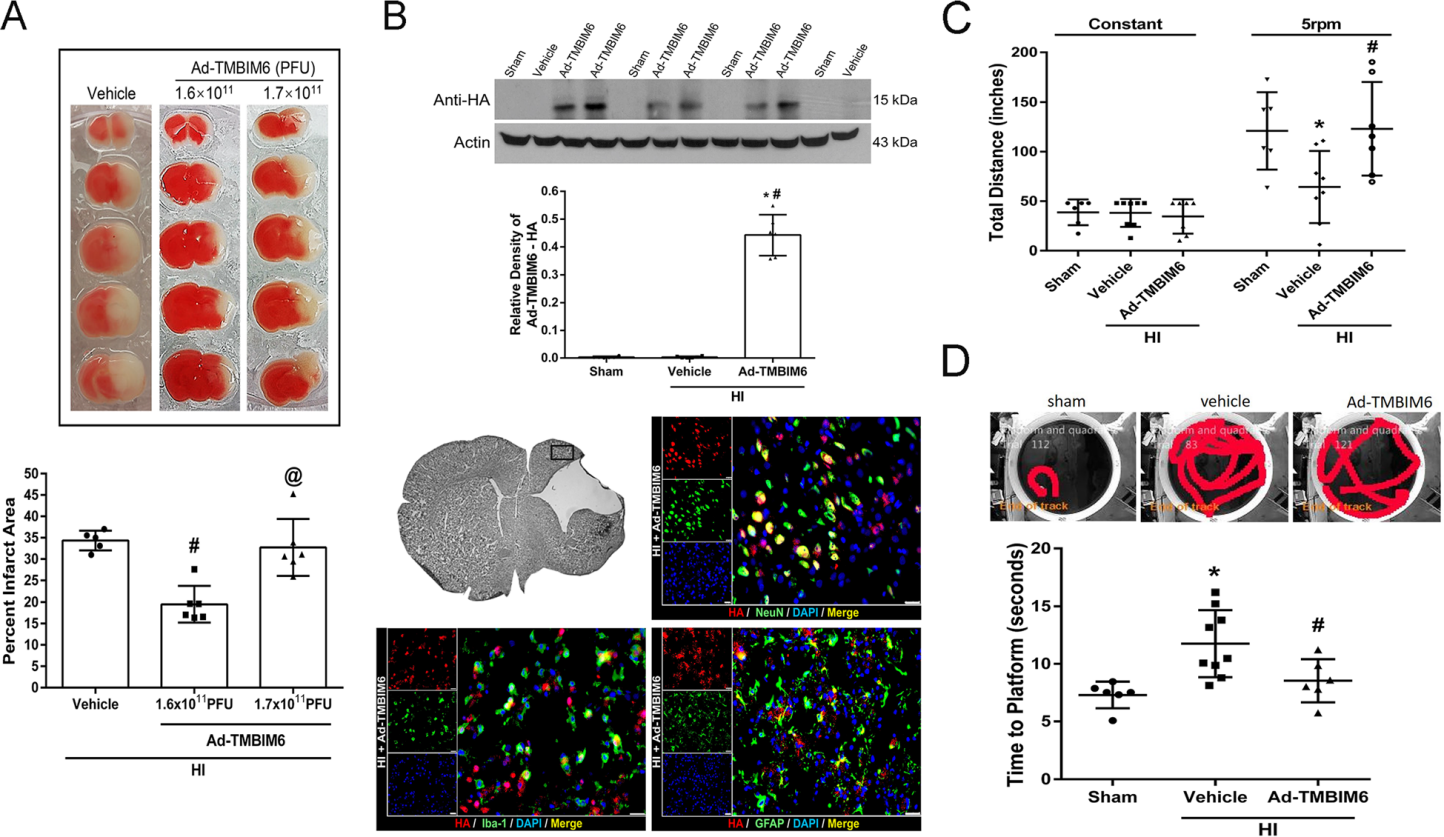
Low dose Ad-TMBIM6 reduced percent infarcted area and improved long-term neurobehavioral outcomes. Quantitative analysis of TTC stained sections for the percent infarcted area at 72 h post-HI (**a**). Quantitative analysis of viral vector present in the brain and representative images of immunofluorescent staining of expression of virus (red) on neurons (green), microglia (green) and astrocytes (green) at 72 h post-HI (**b**) (scale bar = 25 μm). Long-term neurobehavioral tests: total distance in rotarod test (**c**) and time to platform in water maze test (**d**) at 4 weeks post-HI (data expressed as mean ± SD; an asterisk indicates *p* < 0.05 vs sham; number sign *p* < 0.05 vs vehicle; @ *p* < 0.05 vs Ad-TMBIM6 (1.6 × 10^11^ PFU); *n* = 6–9. One-way ANOVA followed by Tukey’s multiple-comparison/or Holm-Sidak post hoc analysis)

Two doses of the viral vector (1.6 × 10^11^ PFU/mL or 1.7 × 1011 PFU/mL) were injected intracerebroventricularly (icv) at 48 h pre-HI, and infarct percentage was measured at 72 h post-HI. TTC staining results indicated that the low dose (1.6 × 10^11^ PFU/mL) viral vector was more effective as it significantly reduced the percent infarcted area when compared to either vehicle or the high dose vector (1.7 × 1011 PFU/mL, *p* < 0.05, Fig. 2a).

Quantification data of western blot bands showed that there was a significantly higher amount of Ad-TMBIM6 present at 72 h post-HI versus the sham or vehicle groups. To further confirm these findings, immunofluorescent staining results indicated high expression of Ad-TMBIM6 in the brain when stained with anti-HA tagged antibody and showed to co-localize with neurons, microglia, and astrocytes (*p* < 0.05, Fig. 2b). There was no staining seen for Ad-TMBIM6 in sham or vehicle (data not shown).

Please refer to Additional file 5 for detailed statistical analysis.

### Ad-TMBIM6 improved long-term neurological outcomes at 4 weeks post-HI

Neurological function was assessed by rotarod and water maze at 4 weeks post-HI. In both behavioral tests, the vehicle group performed significantly worse compared to sham animals. In the rotarod test, the Ad-TMBIM6-treated group showed to significantly improve sensorimotor coordination as displayed by the longer distance traveled, on the rotating rod (*p* < 0.05, Fig. 2c), versus vehicle. In the water maze test, compared to the sham group, the vehicle group demonstrated substantial memory impairment and learning abilities in terms of the time it took to reach the platform (*p* < 0.05, Fig. 2d). However, Ad-TMBIM6 significantly improved both memory and learning function compared to vehicle (*p* < 0.05, Fig. 2d). Please refer to Additional file 5 for detailed statistical analysis.

### Silencing BI-1 or Nrf-2 reversed Ad-TMBIM6’s protective effects at 72 h post-HI

**Fig. 3:**
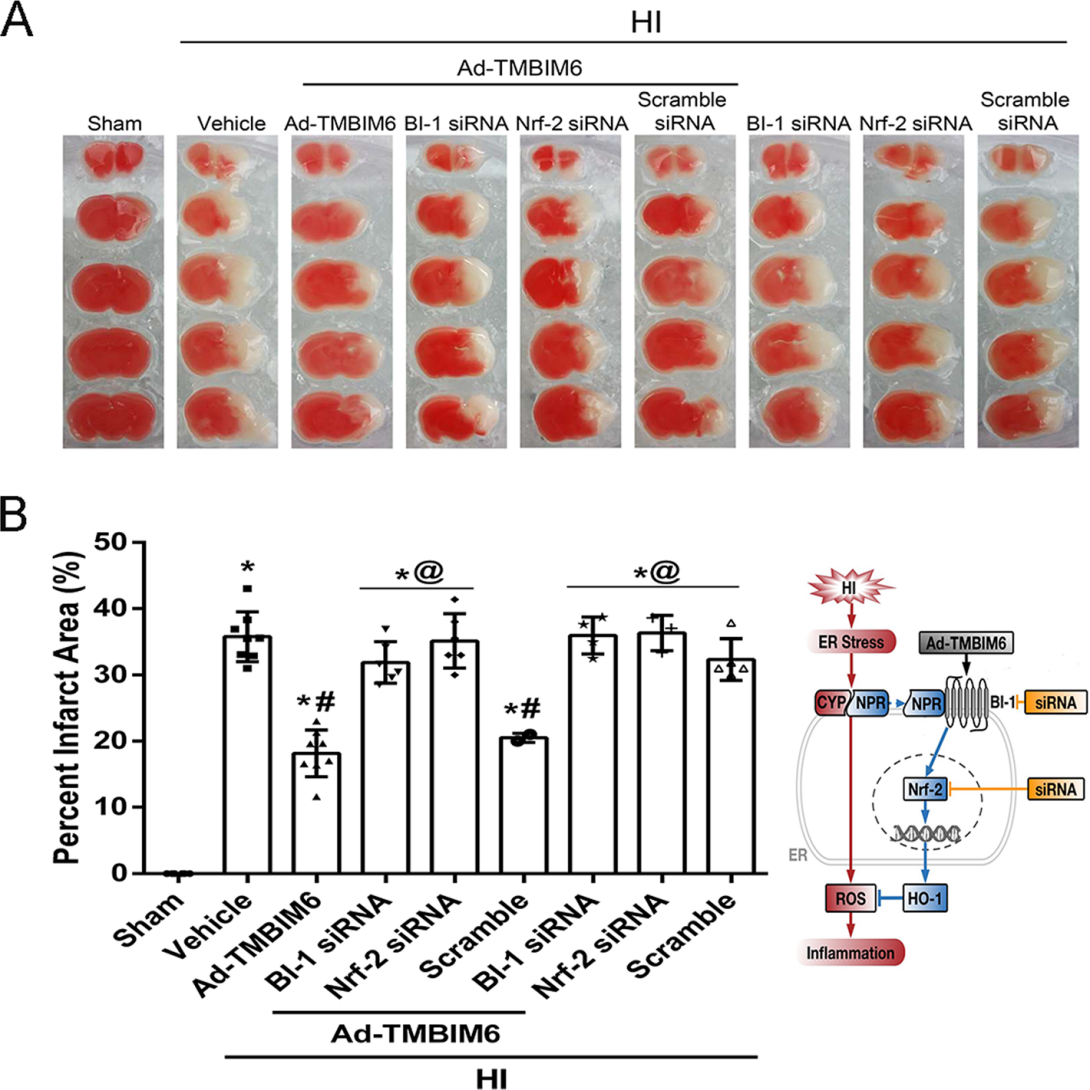
Silencing BI-1 or Nrf-2 increased percent infarcted area at 72 h Post-HI. Quantitative analysis of percent infarcted area after administration of BI-1 or Nrf-2 siRNA (**a**, **b**) (data expressed as mean ± SD; an asterisk indicates *p* < 0.05 vs sham; number sign *p* < 0.05 vs vehicle; @ *p* < 0.05 vs Ad-TMBIM6 or scramble; *n* = 6–8/group using one-way ANOVA followed by Tukey’s multiple-comparison post hoc analysis)

To evaluate the effects of BI-1 or Nrf-2 siRNA on the percent infarcted area, animals were sacrificed at 72 h post-HI and brain samples harvested for TTC staining. Results showed that Ad-TMBIM6 + BI-1siRNA significantly reversed BI-1’s protective effects as did the Ad-TMBIM6 + Nrf-2siRNA group. This is seen from the significant increase in percent infarcted area when compared to the Ad-TMBIM6 only group (*p* < 0.05, Fig. 3a, b). The control group, Ad-TMBIM6 + scramble siRNA, did not exacerbate the damage when compared to Ad-TMBIM6-treated group. Furthermore, BI-1 and Nrf-2 siRNA only groups (without treatment) showed similar percent infarcted areas as compared to HI + vehicle or HI + scramble siRNA groups. Please refer to Additional file 6 for detailed statistical analysis.

### Overexpression of BI-1 disrupted the NPR-CYP complex at 72 h post-HI

**Fig. 4:**
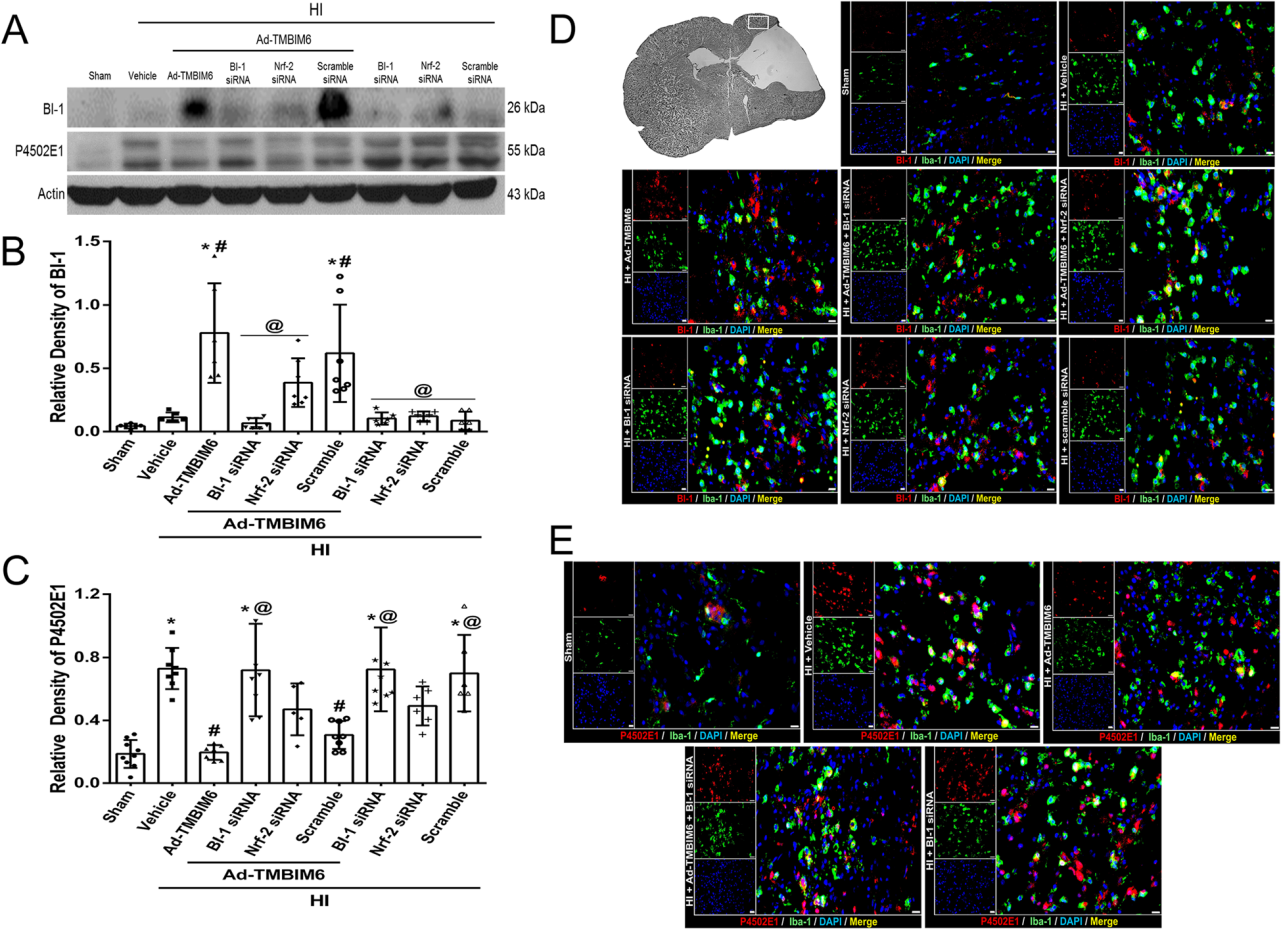
The effects of silencing BI-1 on the NPR-CYP complex at 72 h post-HI. Representative bands of western blot data (**a**). Quantification of western blot bands for BI-1 (**b**) and P4502E1 (**c**) (data expressed as mean ± SD; an asterisk indicates *p* < 0.05 vs sham; number sign *p*< 0.05 vs vehicle; @ *p*< 0.05 vs Ad-TMBIM6 or scramble; *n* = 6/group using one-way ANOVA followed by Tukey’s multiple-comparison post hoc analysis). Immunofluorescence staining of Iba-1 with BI-1 or P4502E1 (**d**, **e**). Green was for microglial staining, red was for BI-1 or P4502E1 staining, and blue was for DAPI. Merge (yellow) showed the co-localization of BI-1 or P4502E1 on microglia (scale bar = 50 μm; *n* = 3/group)

Quantification data showed that BI-1 expression levels were significantly increased in the Ad-TMBIM6-treated and in the Ad-TMBIM6 + scramble siRNA groups, while BI-1 siRNA reversed those effects (*p* < 0.05, Fig. 4a, b). In addition, BI-1 expression levels were low in all three groups (without Ad-TMBIM6) BI-1 siRNA, Nrf-2 siRNA, and scramble.

Ad-TMBIM6 significantly reduced the expression of P4502E1 (*p* < 0.05, Fig. 4c), while silencing BI-1 reversed those effects, as seen from the elevated expression levels of P4502E1 (*p* < 0.05, Fig. 4c). Please refer to Additional file 7 for detailed statistical analysis.

To observe changes in BI-1 and P4502E1 expression levels, as well as co-localization with microglia, after either Ad-TMBIM6 or after silencing of BI-1 and Nrf-2, immunofluorescence staining was performed. Data showed increased expression of BI-1 and co-localization on microglia in Ad-TMBIM6 treatment group and scramble control group compared to sham or vehicle while BI-1 siRNA reversed those effects (Fig. 4d). On the contrary, P4502E1 expression levels were elevated in the vehicle, BI-1 siRNA, and Nrf-2 siRNA groups but not in sham or Ad-TMBIM6-treated groups (Fig. 4e). There was also col-localization of P4502E1 on microglia.

### BI-1 upregulated pNrf-2 expression and induced anti-oxidant enzyme production at 72 h post-HI

**Fig. 5:**
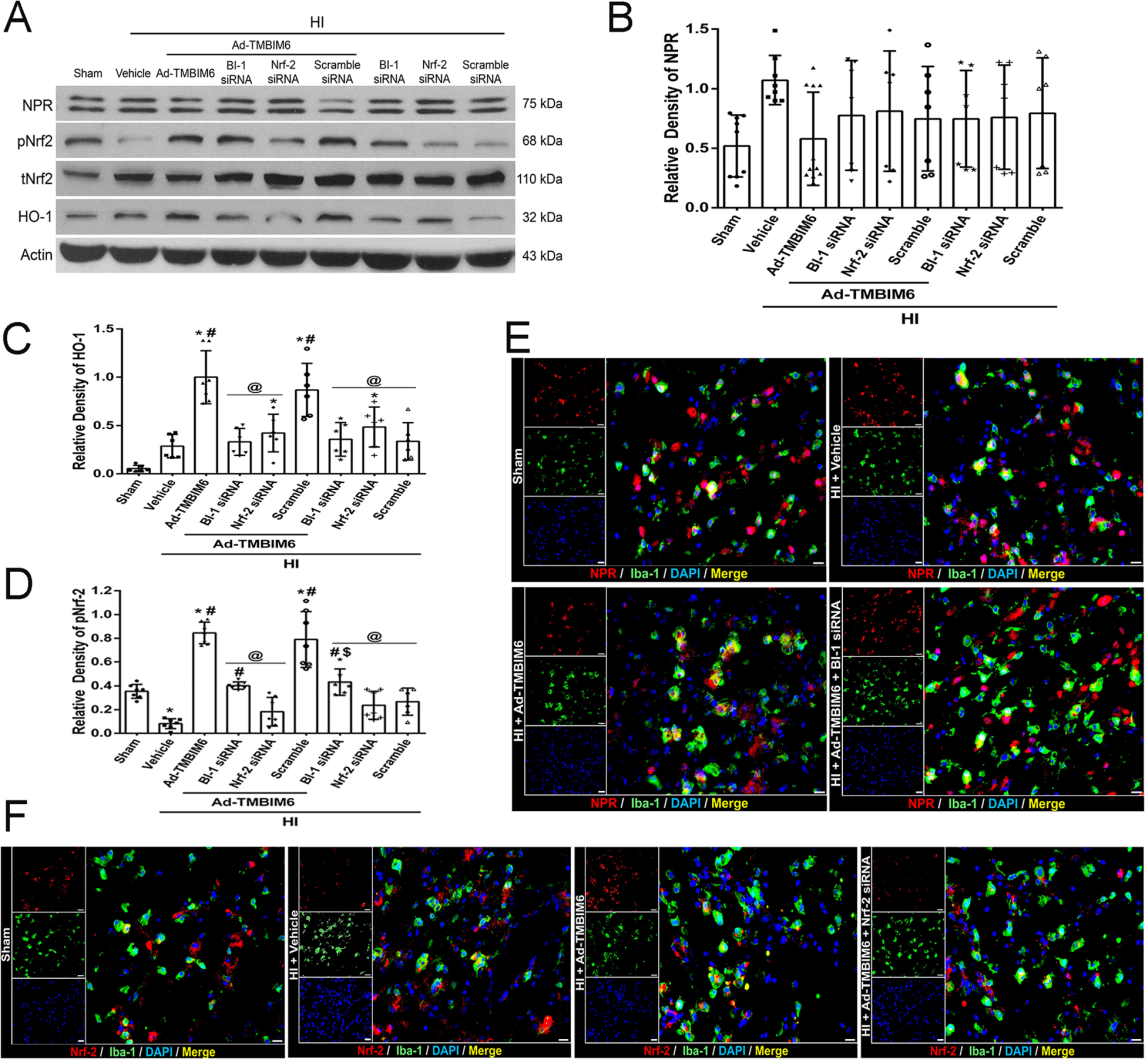
BI-1’s role in the Nrf-2 signaling pathway at 72 h post-HI. Representative picture of western blot data (**a**). Quantification of band intensities for NPR (**b**), HO-1 (**c**), and pNrf-2 (**d**). Immunofluorescence staining of Iba-1 with NPR or Nrf-2 (**e**, **f**). Green was for microglial staining, red was for NPR or Nrf-2 staining, and blue was for DAPI. Merge showed the co-localization of NPR or Nrf-2 on microglia (scale bar = 50 μm; *n* = 3/group). Data expressed as mean ± SD; an asterisk indicates *p* < 0.05 vs sham; number sign *p* < 0.05 vs vehicle; @ *p* < 0.05 vs Ad-TMBIM6 or scramble; *n* = 6/group using one-way ANOVA followed by Tukey’s multiple-comparison post hoc analysis

After treatment with Ad-TMBIM6, the expression levels of pNrf-2 were significantly increased compared to the vehicle. Either silencing BI-1 or Nrf-2 with siRNAs reversed those effects (*p* < 0.05, Fig. 5a, c). An increase in Nrf-2 levels was followed by an increase in the antioxidant enzyme, HO-1, in the Ad-TMBIM6 group when compared to vehicle (*p* < 0.05, Fig. 5a, d). Furthermore, inhibition of BI-1 or Nrf-2 significantly decreased HO-1 levels and its ability to block ROS. Please refer to Additional file 7 for detailed statistical analysis.

Double immunofluorescence staining was performed to detect co-localization of NPR or Nrf-2 with microglia (Iba-1) in the presence of Ad-TMBIM6, BI-1 siRNA, or Nrf-2 siRNA. Fluorescent staining showed that although NPR co-localized with microglia, its expression levels were unchanged among groups (Fig. 5e). On the contrary, Ad-TMBIM6 increased Nrf-2 expression and co-localization with microglia compared to sham or vehicle, while Nrf-2 siRNA abolished that effect (Fig. 5f).

### BI-1 overexpression attenuated ROS production and inflammation at 72 h post-HI

**Fig. 6:**
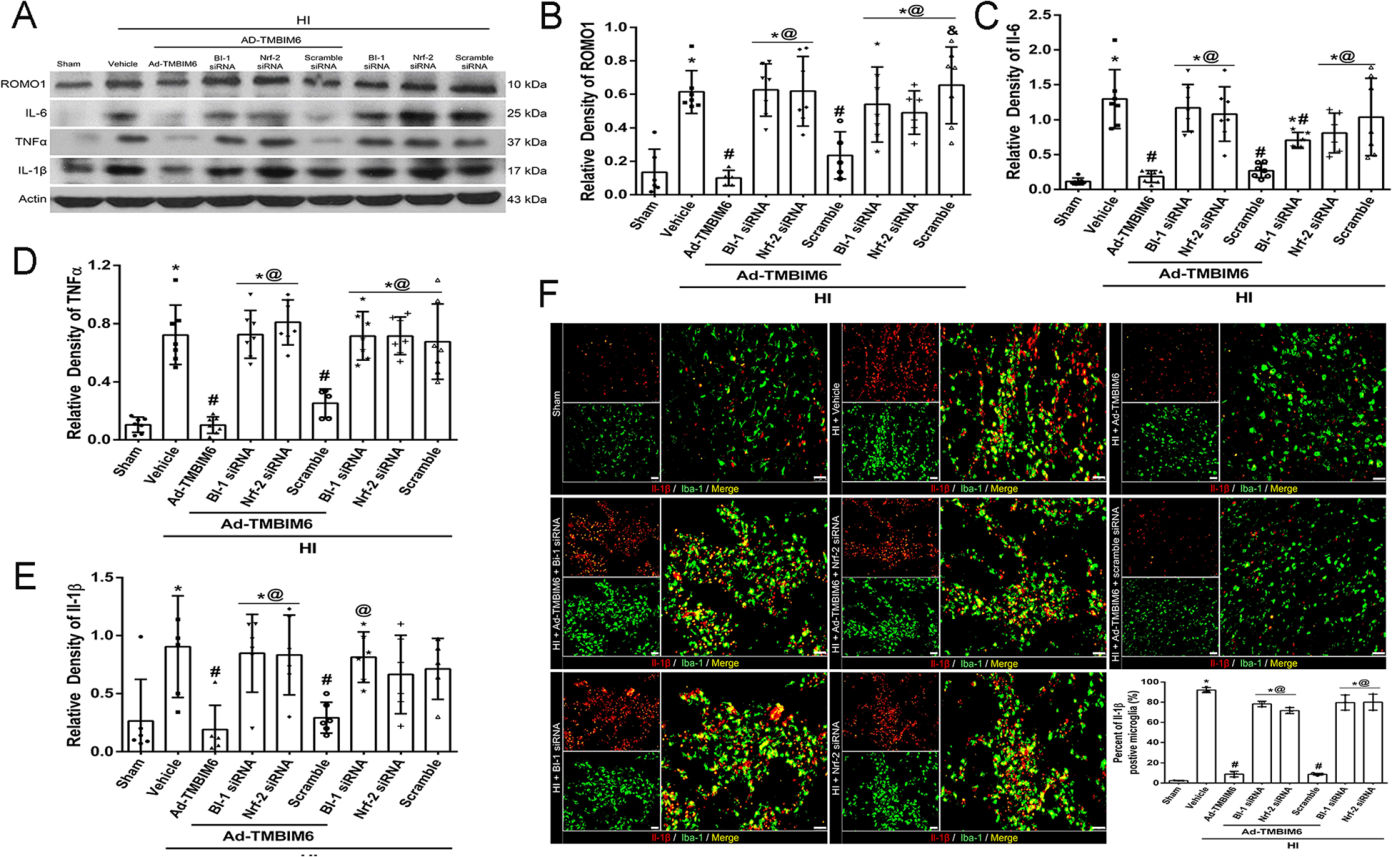
Overexpression of BI-1 attenuated ROS production and inflammation at 72 h post-HI. Representative picture of western blot bands (**a**). Quantification analysis of band intensities for ROMO1 (**b**), IL-6 (**c**), TNFα (**d**), and IL-1β (**e**). Immunofluorescent staining and quantification of the number of positively stained cells of Iba-1 with the inflammatory marker, IL-1β, in the peri-infarcted region (**f**). Green was for microglial staining and red was for IL-1β. Merge showed the co-localization of IL-1β on microglia. Scale bar 50 μm; *n* = 3/group. Data expressed as mean ± SD; an asterisk indicates *p* < 0.05 vs sham; number sign *p* < 0.05 vs vehicle; @ *p* < 0.05 vs Ad-TMBIM6 or scramble; *n* = 6/group using one-way ANOVA followed by Tukey’s multiple-comparison post hoc analysis

To evaluate whether overexpression of BI-1 with Ad-TMBIM6 could inhibit ROS production and subsequently attenuate inflammation, western blot was done to quantify ROMO1 (a modulator that induces production of ROS), IL-6, TNFα, and IL-1β expression levels. Data showed that the Ad-TMBIM6 treatment group significantly reduced ROMO1 levels (*p* < 0.05, Fig. 6a, b) and attenuated pro-inflammatory markers, IL-6, TNFα, and IL-1β (*p* < 0.05, Fig. 6c–e). In addition, the administration of BI-1 siRNA and Nrf-2 siRNA reversed Ad-TMBIM6’s protective effects as seen from the significantly higher upregulation of pro-inflammatory mediators and ROS. Please refer to Additional file 8 for detailed statistical analysis.

Since HI results in the induction of inflammation, we stained for IL-1β (a pro-apoptotic marker) with microglia. The counting of positively stained cells for IL-1β with microglia showed a higher percentage of IL-1β on microglia in the vehicle, BI-1 siRNA, and Nrf-2 siRNA groups compared to sham, Ad-TMBIM6, or scramble group (*p* < 0.05, Fig. 6f; scale bar 50 μm). Please refer to Additional file 9 for detailed statistical analysis.

**Fig. 7:**
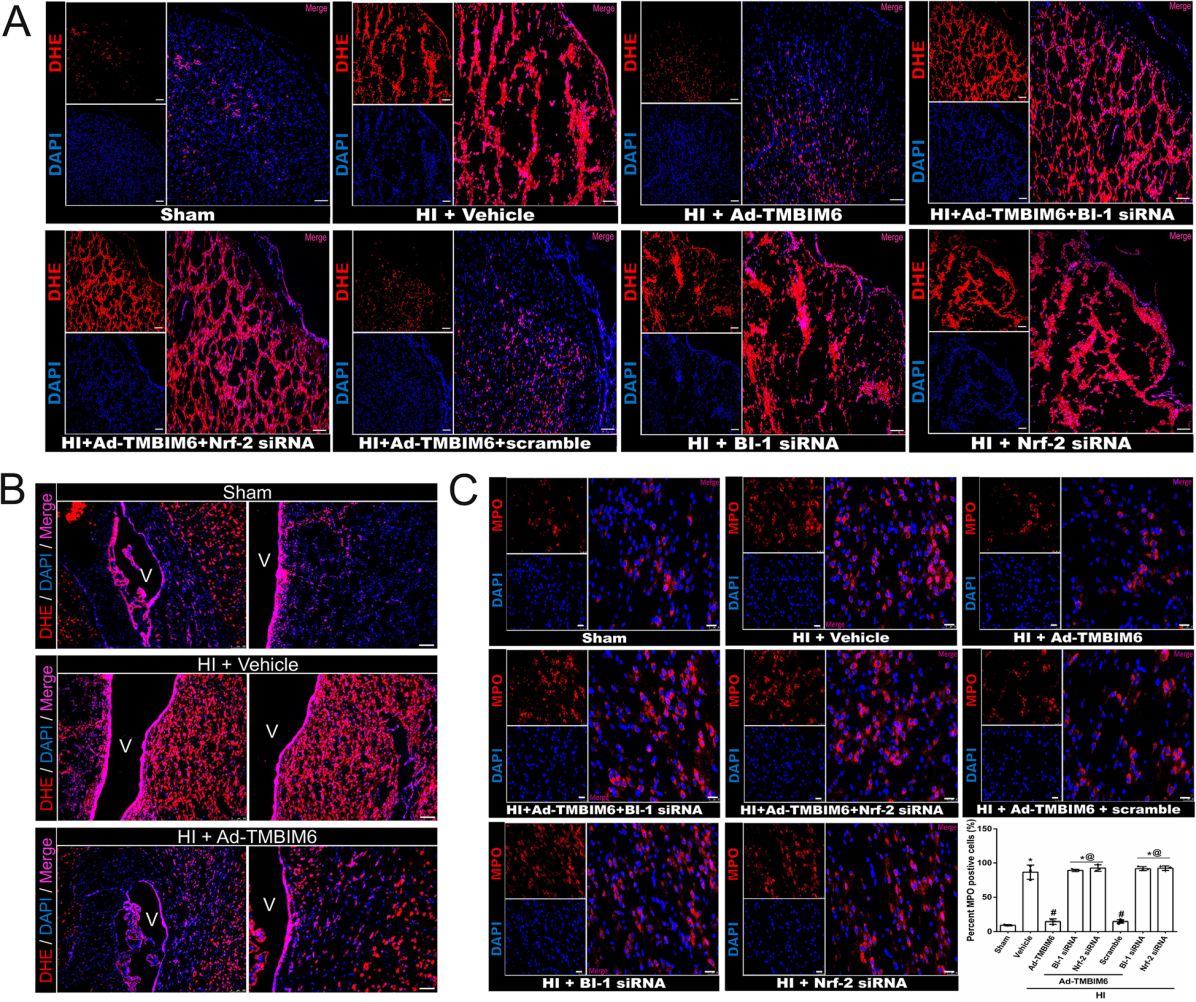
Fluorescent staining demonstrated BI-1’s ability to reduce ROS and attenuate inflammation at 72 h post-HI. DHE staining for ROS positive cells in the cortex (**a**). DHE staining around ventricles for ROS positive cells (**b**). Immunofluorescence and MPO-positive cell counting in peri-infarcted region (**c**). Red was for ROS or MPO staining and blue was for DAPI. Merge showed the number of positively stained cells (**a**–**c**) (for panel **a**, scale bar = 50 μm; for panel **b**, scale bar = 100 μm; for panel **c**, scale bar = 25 μm; *n* = 3/group)

To evaluate ROS production, we stained brain slices with dihydroethidium (DHE) dye. DHE is possibly the most specific and least problematic dye for detection of superoxide radicals. Cells with high ROS accumulation will have a red fluorescent light. Our data revealed a higher number of red positively stained cells in vehicle and siRNA groups versus sham, Ad-TMBIM6, and control group in the cortex region (Fig. 7a).

Furthermore, we also stained for ROS around the ventricles and observed trends similar to the cortex region (Fig. 7b). In addition, to determine whether ROS accumulation is correlated with increased inflammation, we stained for myeloperoxidase (MPO; a marker that shows the inflammatory activity) and detected significantly higher expression of MPO positively stained cells in vehicle and siRNA groups compared to sham or Ad-TMBIM6-treated group (*p* < 0.05, Fig. 7c), thus showing both a correlation between an increase in ROS production with an increase in inflammation, as well as BI-1’s ability to inhibit ROS production and the increase in inflammation independently. Please refer to Additional file 9 for detailed statistical analysis.

### To investigate BI-1’s effects on NPR-CYP anti-inflammatory pathway in an in vitro OGD model

**Fig. 8:**
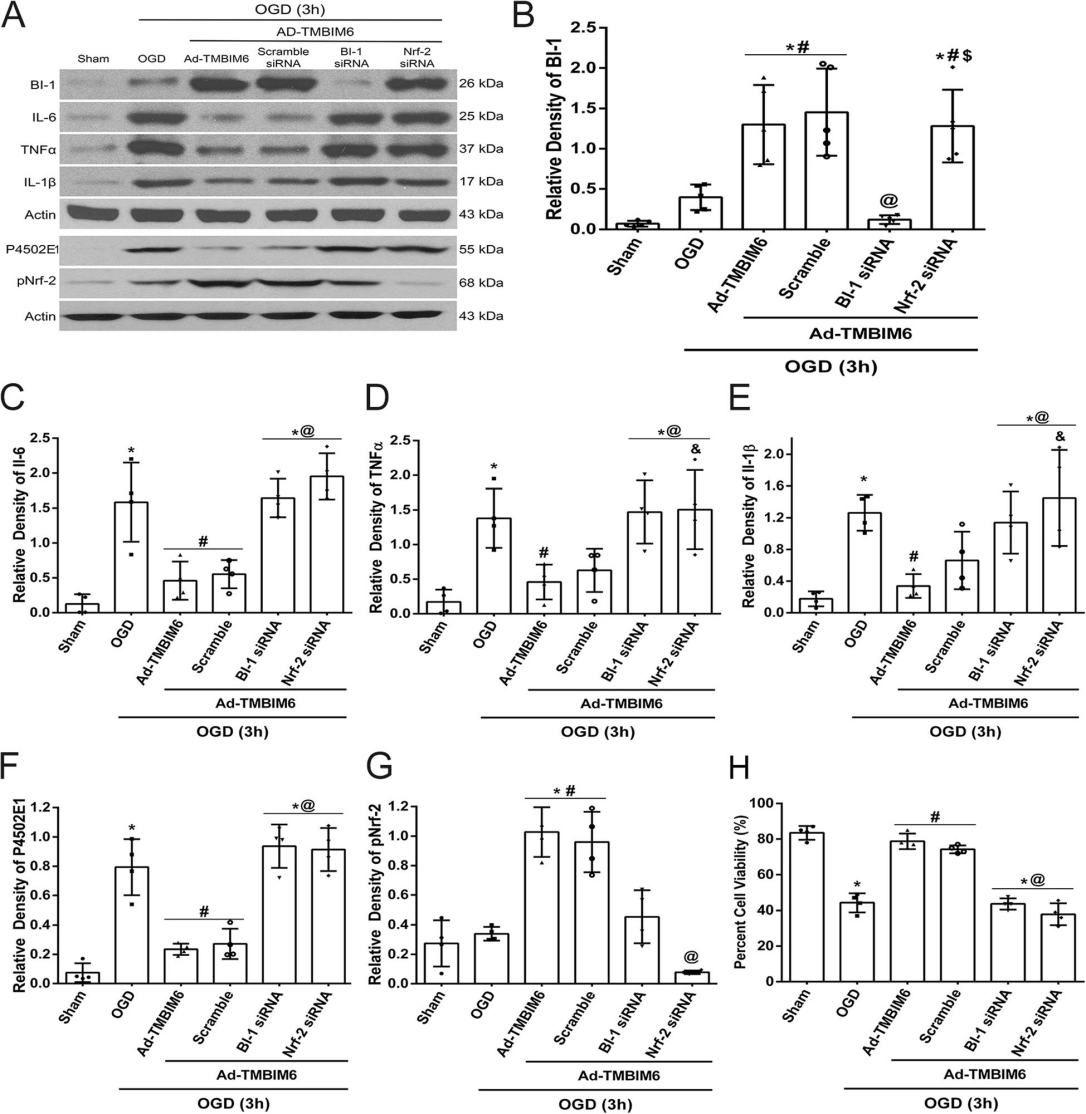
BI-1 exerts its anti-inflammatory effects via inhibition of P4502E1 in an in vitro OGD model. Representative picture of western blot bands (**a**). Quantification analysis of BI-1, IL-6, TNFα, IL-1β, P4502E1, pNrf-2 (**b**–**g**) and percent cell viability (**h**). Data represent ± SD; an asterisk indicates *p* < 0.05 vs control; number sign *p* < 0.05 vs vehicle; @ *p* < 0.05 vs Ad-TMBIM6 and scramble; dollar sign *p* < 0.05 vs BI-1 siRNA; *n* = 4–5/group using one-way ANOVA followed by Tukey’s multiple-comparison post hoc analysis

As BI-1 has been previously reported to disrupt the NPR-CYP complex as well as activate Nrf-2 resulting in inhibition of ROS production and subsequent attenuation of inflammation, we employed an in vitro OGD model using a microglial cell line and primary microglia cell culture (Additional files 2 and 3) to test the specificity of BI-1 related attenuation of ROS-induced inflammation in microglia. To test BI-1’s pathway, we administered a BI-1 siRNA to knockdown BI-1 expression levels and Nrf-2 siRNA to silence Nrf-2 expression. Data showed that the administration of Ad-TMBIM6 vector significantly upregulated BI-1 levels in cells, both in treated and scramble control group, while BI-1 siRNA group reversed those effects; Nrf-2 siRNA did not affect BI-1’s levels (*p* < 0.05, Fig. 8a, b). Intervening at either BI-1 level or Nrf-2 level with siRNAs significantly reversed BI-1’s protective effects as seen from the increased expression levels of inflammatory markers, IL-6, TNFα, and IL-1β, when compared to Ad-TMBIM6-treated group, sham, or scramble groups (*p* < 0.05, Fig. 8c–e). Our data demonstrated that Ad-TMBIM6 inhibited P4502E1, the primary source of ROS production, while simultaneously upregulating pNrf-2 levels (*p* < 0.05, Fig. 8f, g). Silencing BI-1 or Nrf-2 reversed those effects. Percent cell viability data showed that Ad-TMBIM6 was able to significantly improve the percent of viable cells while the knockdown of BI-1 and Nrf-2 reversed those effects (*p* < 0.05, Fig. 8h). Please refer to Additional file 10 for detailed statistical analysis.

## Discussion

The endoplasmic reticulum (ER) is a major organelle that has an essential role in multiple cellular processes, such as the control of correct protein folding and function [47, 50]. Hypoxia-ischemia, oxidative stress, calcium disturbances, and inhibition of protein glycosylation may contribute to a disruption in ER homeostasis and lead to stress [50]. Cells respond to ER stress by activating several pathways, including promoting the ability of proteins to refold correctly, inhibiting protein translation, increasing protein degradation, stimulating the transcription of genes, and enabling self-repair mechanisms [50]. All these processes are referred to as the unfolded protein response (UPR) which under prolonged activation result in cell death [47].

Bax Inhibitor-1 (BI-1) is a conserved evolutionary protein that resides on the intracellular membrane of the ER [12]. BI-1 was first named as Bax Inhibitor due to its ability to suppress cell death in yeast [49]. More recently, it is also referred to as TMBIM6 as it is part of the transmembrane Bax Inhibitor-1 containing motif 6 family [19]. Overexpression of BI-1 has been demonstrated to play a protective role in ER stress-induced cell death, and to a lesser extent in other ER related stresses, such as oxidative stress and inflammation. In the present study, we first measured endogenous expression levels of BI-1 and its downstream proteins. Our results showed that endogenous expression of P4502E1 (CYP2E1), a member of the MMO system and a major inducer of ROS generation, increased in a time-dependent manner after HI and remained elevated through 72 h post HI. This is expected as after HI injury, there is a significant increase in ROS production that is regulated mainly through P4502E1 (Fig. 1a, d). Hence, levels of P4502E1 increase during times of injury. On the contrary, BI-1’s role is to reduce ROS by disrupting P4502E1. In our results, we demonstrated an initial increase in BI-1 expression; however, at 72 h post HI, BI-1 expression levels significantly declined (Fig. 1a, b). This initial increase in BI-1 can be explained as a protective response to the injury. BI-1 levels increase with the attempt to reverse the damage caused by HI injury. However, the endogenous levels are not enough to reverse the damage and BI-1 declines significantly by 72 h as seen from our data. Therefore, the main goal in our study was to significantly increase BI-1 levels in the brain, using exogenous means, to be able to reverse damage caused after HI injury.

To demonstrate BI-1’s protective effects after hypoxic-ischemic (HI) injury in the neonatal rat, we overexpressed BI-1 using Adenoviral-TMBIM6 (Ad-TMBIM6) vector. We tested two doses and showed that low dose Ad-TMBIM6 vector significantly reduced the percent infarcted area (Fig. 2a) and improved long-term neurobehavioral outcomes (Fig. 2c–d). In addition, we quantified the amount of viral vector present in the brain using western blot and showed robust expression of Ad-TMBIM6 at 72 h post-HI (Fig. 2b).

ER stress is associated with the production of reactive oxygen species (ROS) through oxidative protein folding by the MMO system, composed of NADPH-P450 reductase (NPR) and cytochrome P450 (CYP) members such as P4502E1 [27, 28, 32]. It has been shown that BI-1 overexpressing cells can regulate UPR induction and inhibit ROS accumulation under ER stress [15], thus making BI-1 a crucial regulator of ROS inhibition. Cells respond to ROS by activating genes to encode antioxidative stress enzymes. A key transcription factor activated is nuclear factor erythroid 2-related factor 2 (Nrf-2), which regulates the production of several cytoprotective enzymes, such as heme oxygenase-1 (HO-1), a potent inhibitor of ROS [15, 17]. Studies showed that in BI-1 overexpressing cells, inhibition of HO-1 attenuated BI-1-mediated protection against ER stress [17]. However, the role of HO-1 in BI-1’s protective mechanism is mostly unknown and debatable.

Some studies have shown that HO-1 was unaffected in BI-1 deficient mice embryonic fibroblast cells [24]. In contrast, BI-1 overexpressed cells showed significant inhibition of P4502E1 expression [15] and suppression of ROS production in human embryonic kidney cells [16]. The P4502E1 member of the CYP ER heme proteins is a major contributor to ROS production. It acts by metabolizing and activating substrates into more toxic products, thus not only increasing ROS production but also stimulating inflammatory cascades and ultimately worsening HI pathology. Previous studies have shown P4502E1 to be upregulated during ER stress [15–18], thus playing an essential role in HI pathology.

In addition, other studies showed that P4502E1-induced oxidative stress played a role in the translocation of Nrf-2 to the nucleus followed by the upregulation of HO-1. Moreover, increased HO-1 levels may be dependent on P4502E1 [9], perhaps attempting to counteract P4502E1 effects and act as a survival signal. The same study showed that the inhibition of P4502E1 significantly reduced ROS generation in an acute kidney injury model [43]. The relationship between P4502E1 and HO-1 needs further research in order to fully understand the specifics covering the apparent connection. Since P4502E1 is a major regulator of ROS production and that Nrf-2 has a role in the response against P4502E1, inhibition of P4502E1 coupled with activation of Nrf-2 is a promising therapeutic target.

Although we are now beginning to understand the importance of BI-1 in the cell and in human physiology, its function and signaling mechanisms remain unknown. Given the importance of P4502E1 and Nrf-2, finding a key molecule to regulate both is of great interest. Here we identified BI-1 as one such potential molecule which may protect the cell against ER stress-induced ROS production as well as the subsequent increase in inflammatory response via two possible mechanisms. First, it may interact with NPR, thus destabilizing the NPR-CYP complex, which directly inhibits the formation of ROS by reducing the activity of P4502E1 [15]. BI-1 may interact through its C-terminus with NPR and to a lesser extent with P4502E1 [15]. This interaction induces destabilization of the NPR-CYP complex, thus blocking electron transfer and ROS production [32]. A recent study showed that BI-1 overexpressing cells caused P4502E1 degradation, leading to ER stress suppression, and subsequent reduction in ROS [18]. We observed similar findings from our western blot results; overexpression of BI-1 in the neonatal rat significantly upregulated BI-1 levels in the brain, while simultaneously reducing P4502E1 expression levels (Fig. 4a–c). Silencing BI-1 reversed those effects while silencing Nrf-2 did not significantly change P4502E1 levels, which indicates Nrf-2 to be downstream of P4502E1. This data was further supported by our IHC staining that demonstrated across groups that as BI-1 expression increased, P4502E1 decreased; they have converse expression patterns throughout (Fig. 4d, e). Our western blot data also demonstrated a decrease in BI-1 expression levels after silencing Nrf-2 with siRNA (Fig. 4a). This may be explained due to BI-1’s multimodal properties and hence targeting multiple signaling pathways. In our study, we focused on BI-1 and its role on the CYP-NPR complex and Nrf-2 signaling. Specifically, we showed that BI-1 may upregulate Nrf-2, an anti-oxidative molecule, thus attenuating ROS production and subsequent inflammation. However, BI-1 has other roles such as being able to reduce Bax via direct interaction with Bcl-2 [45, 49]. This interaction with Bcl-2 showed to increase its levels while decreasing the pro-apoptotic protein, Bax [45, 49]. Like BI-1, Nrf-2 levels may be affected by different signaling pathways as well. In this case, as previous studies have shown an interaction between BI-1 and Bcl-2, studies have also shown a direct connection between Nrf-2 and Bcl-2 [23, 29, 30, 39, 51]. Specifically, Nrf-2 has been shown to bind to Bcl-2 ARE and regulate the expression and induction of the Bcl-2 gene [30]. Since both Nrf-2 and BI-1 have a direct interaction with Bcl-2, this may explain as to why the decrease in Bcl-2 levels, due to knockdown of either Nrf-2 or BI-1, will indirectly affect each other’s expression levels. In our study, we knocked down Nrf-2, using siRNA, and saw a decrease in BI-1 expression. This result may be explained, as mentioned above, by the fact that knocking down Nrf-2 affects Bcl-2 levels (Bcl-2 decreases) and since BI-1 and Bcl-2 have a direct interaction as well, it will also affect BI-1 expression levels acting like a negative feedback loop, which resembles the decrease we observed in our experiment (Fig. 4).

Second, BI-1 may upregulate pNrf-2 which in turn triggers HO-1 thus inhibiting ROS production and attenuating inflammation. Lee et al. showed that overexpression of BI-1 in cells increased Nrf-2 transcription factor, which then translocated to the nucleus where it stimulated the production of anti-oxidant enzymes, HO-1. HO-1 is known to block ROS production and accumulation thereby promoting cell survival [17]. Similar to Lee et al., we found that BI-1 overexpression, after neonatal HI, significantly upregulated pNrf-2 (Fig. 5c) and HO-1 (Fig. 5d) while silencing of either BI-1 or Nrf-2 with siRNAs reversed those effects. In addition, our IHC staining showed changes in Nrf-2 expression patterns among groups that correlated with our western blot findings (Fig. 5f). Furthermore, inhibition of P4502E1 with activation of Nrf-2 and HO-1 was linked with a reduction in ROS accumulation and the subsequent release of pro-inflammatory mediators. Here, we used a ROS marker, Reactive Oxygen Species Modulator 1 (ROMO1), which is a protein-coding gene responsible for ROS generation. There was a significant reduction in ROMO1 expression levels (Fig. 6a, b) as well as in IL-6, TNF-α, and IL-β (Fig. 6c, d). Staining for IL-1β on microglia demonstrated a significantly higher expression and co-localization in the vehicle, BI-1 siRNA, and Nrf-2 siRNA groups compared to sham or Ad-TMBIM6-treated group (Fig. 6f). These results were confirmed to be via BI-1-induced inhibition of P4502E1 or activation of Nrf-2 as either inhibition of BI-1 or Nrf-2 reversed those effects. To further examine BI-1’s inhibitory role on ROS accumulation, we used a dihydroethidium (DHE) dye to detect ROS production [31]. The dye binds with the superoxide anions, thus illuminating a red fluorescent image which is an indication of ROS presence. Our data indicated a higher amount of ROS accumulation in vehicle and siRNA groups versus sham or Ad-TMBIM6-treated groups, in both cortex region and around ventricles, while overexpression of BI-1 reversed those effects (Fig. 7a, b). To detect whether a reduction in ROS was associated with a reduction in inflammation, we performed immunofluorescence staining for myeloperoxidase (MPO). A similar pattern was observed in the MPO staining where Ad-TMBIM6 significantly reduced MPO positively stained cells compared to vehicle or siRNA groups (Fig. 7c), thus indicating a correlation between a reduction in ROS and the subsequent attenuation of inflammatory processes.

## Conclusions

In summary, the overexpression of BI-1 attenuated inflammation and demonstrated to promote cell survival by increasing the production of antioxidant enzymes as well as destabilizing the complex responsible for ROS production. This was observed as either silencing BI-1 or Nrf-2 reversed BI-1’s protective effects by altering P4502E1, Nrf-2, and HO-1 expressions. This is indicative of BI-1’s ability to interact with the NPR-CYP complex and induce dissociation, thus disrupting the electron flow, as a physical association between NPR and CYP is required for the production of ROS. Furthermore, BI-1 was shown to upregulate pNrf-2 and HO-1 directly. These two mechanisms play a major role in BI-1’s anti-inflammatory effects. In addition, our in vivo findings were translated to an in vitro OGD model to validate this pathway in microglial cells (Fig. 8, Additional files 2 and 3).

This study expands the current evidence on BI-1 as a protective protein and advances present in vitro studies to in vivo stroke model. A major challenge with any mechanism design lies in the existence of several parallel pathways that may act at the same time. Thus, focusing on only one may not be ideal for preserving cell survival. BI-1’s multimodal properties suggest that it can target a wide array of pathophysiological consequences after HI, thus making it an ideal therapeutic candidate.

## Additional files

Additional file 1: Experimental Design**.** Representative figure showing experimental design and animal groups. HI, Hypoxia- Ischemia; IHC, immunohistochemistry; DHE, Dihydroethidium. (TIF 2430 kb)

Additional file 2: Ad-TMBIM6 attenuates inflammation in primary microglial cells after OGD. Representative western blot bands of BI-1, P4502E1, IL-6 and IL-1β (A). Immunofluorescent staining of Iba-1 with P4502E1, BI-1 or pNrf-2 (B-D). (Green was for microglial staining, Red was for P4502E1, BI-1 or pNrf-2. Merge showed the co-localization of P4502E1, BI-1 or pNrf-2 on microglia. Scale bar 50 μm). (TIF 3861 kb)

Additional file 3: Ad-TMBIM6 attenuates ER stress receptors’ signaling in primary microglial cells after OGD. Representative western blot bands of pIRE1α, XBP1, pPERK and peIF2α (A). Immunofluorescent staining of Iba-1 with pIRE1α or pPERK (B-C). (Green was for microglial staining, Red was for pIRE1α or pPERK. Merge showed the co-localization of pIRE1α or pPERK on microglia. Scale bar 50 μm). (TIF 2942 kb)

Additional file 4: Statistical Analysis for Endogenous Protein Expression Levels post-HI. Representative table showing detailed statistical analysis for the endogenous expression levels of BI-1, NPR, P4502E1, pNrf-2 and HO-1. (Data expressed as mean +/− SD; *p < 0.05 vs sham; # p<0.05 vs 6 h, @ *p* < 0.05 vs 24 h; *n* = 4, using one-way ANOVA followed by Tukey multiple-comparison post hoc analysis). (XLSX 12 kb)

Additional file 5: Statistical Analysis for Percent Infarcted Area and Long-term studies. Representative table showing detailed statistical analysis for dose study, viral vector present in the brain and long-term neurobehavior studies. (Data expressed as mean +/− SD; *p < 0.05 vs sham; # p<0.05 vs vehicle, @ *p* < 0.05 vs Ad-TMBIM6 (1.6 × 10^11^ PFU), *n* = 6. One-way ANOVA followed by Tukey multiple-comparison post hoc analysis). (For long-term studies: *p<0.05 vs sham; #p<0.05 vs vehicle; *n* = 6–9. One-way ANOVA followed by Tukey multiple-comparison/or Holm-Sidak post hoc analysis). (XLSX 11 kb)

Additional file 6: Statistical Analysis for Percent Infarcted Area with Intervention groups at 72 h post-HI. Representative table showing detailed statistical analysis for viral vector present in the brain and the percent infarcted area seen with different intervention groups. (Data expressed as mean +/− SD; *p < 0.05 vs sham; #*p* < 0.05 vs vehicle; @*p* < 0.05 vs Ad-TMBIM6 or scramble; *n* = 6–8/group using one-way ANOVA followed by Tukey multiple-comparison post hoc analysis). (XLSX 10 kb)

Additional file 7: Statistical Analysis for Expression Levels of BI-1 Pathway Proteins at 72 h post-HI. Representative table showing detailed statistical analysis from western blot experiments measuring the levels of pathway proteins. (Data expressed as mean +/− SD; *p < 0.05 vs sham; #*p* < 0.05 vs vehicle; @*p* < 0.05 vs Ad-TMBIM6 or scramble; *n* = 6/group using one-way ANOVA followed by Tukey multiple-comparison post hoc analysis). (XLSX 16 kb)

Additional file 8: Statistical Analysis of Inflammatory Protein Expression Levels at 72 h post-HI. Representative table showing detailed statistical analysis from western blot experiments measuring the levels of pathway proteins. (Data expressed as mean +/− SD; *p < 0.05 vs sham; #*p* < 0.05 vs vehicle; @*p* < 0.05 vs Ad-TMBIM6 or scramble; *n* = 6/group using one-way ANOVA followed by Tukey multiple-comparison post hoc analysis). (XLSX 15 kb)

Additional file 9: Statistical analysis of IL-1β and MPO positive cells at 72 h post-HI. Representative table showing detailed statistical analysis from immunohistochemistry experiments measuring the expression of IL-1β with microglia and MPO. (Data expressed as mean +/− SD; *p < 0.05 vs sham; #*p* < 0.05 vs vehicle; @*p* < 0.05 vs Ad-TMBIM6 or scramble; *n* = 6/group using one-way ANOVA followed by Tukey multiple-comparison post hoc analysis). (XLSX 11 kb)

Additional file 10: Statistical Analysis for Expression Levels of BI-1 pathway proteins in an in vitro OGD model. Representative table showing detailed statistical analysis from western blot experiments measuring the levels of BI-1, P4502E1, pNrf-2 and inflammatory markers. (Data represent +/− SD; *p < 0.05 vs control; #*p* < 0.05 vs vehicle; @*p* < 0.05 vs Ad-TMBIM6 and scramble; $*p* < 0.05 vs BI-1 siRNA; *n* = 4–5/group using one-way ANOVA followed by Tukey multiple-comparison post hoc analysis). (XLSX 14 kb)

## Data Availability

All data generated during the current study are available from the corresponding author upon request.

## Abbreviations

Ad-TMBIM6: Adenoviral-Transmembrane Bax inhibitor Motif Containing 6
BI-1: Bax Inhibitor-1
CYP: Cytochrome P450
DHE: Dihydroethidium
ER: Endoplasmic reticulum
HI: Hypoxia-ischemia
HIE: Hypoxic-ischemic encephalopathy
HO-1: Heme oxygenase-1
ICV: Intracerebroventricular
IHC: Immunohistochemistry
IL-1β: Interleukin-1beta
IL-6: Interleukin-6
MMO: Microsomal monooxygenase
MOI: Multiplicity of infection
MPO: Myeloperoxidase
NPR: NADPH-P450 reductase
Nrf-2: Nuclear factor erythroid 2-related factor 2
OGD: Oxygen glucose deprivation
ROMO1: Reactive Oxygen Species Modulator 1
ROS: Reactive oxygen species
TNFα: Tumor Necrosis Factor alpha
UPR: Unfolded protein response
