# Supplementary figures and images for: Viral-mediated gene delivery of TMBIM6 protects the neonatal brain via disruption of NPR-CYP complex coupled with upregulation of Nrf-2 post-HI
Source: J Neuroinflammation. 2019 Aug 31;16:174. doi: 10.1186/s12974-019-1559-4 (PMC6717394; doi:10.1186/s12974-019-1559-4)

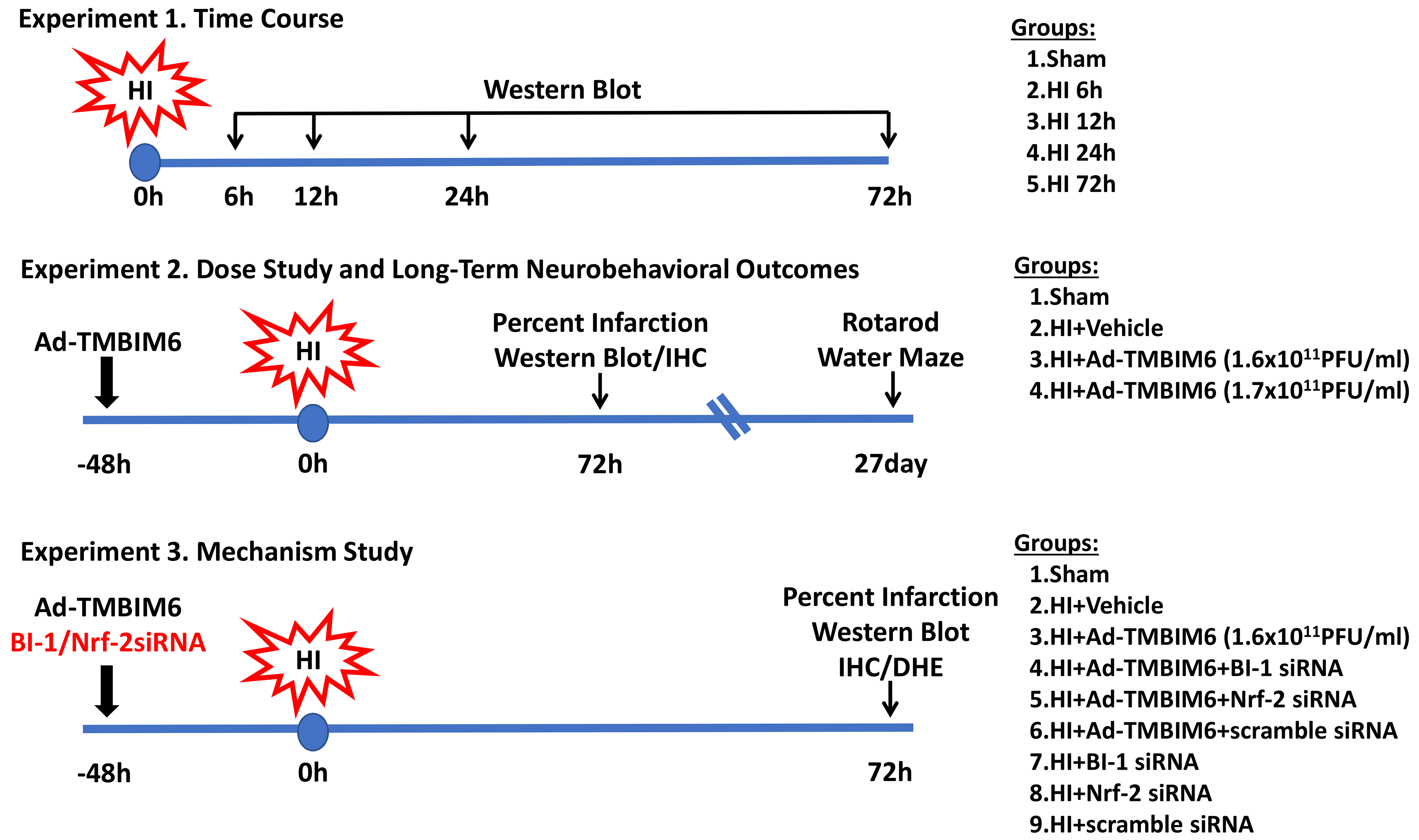

Supplement: Supplementary file 1 — Experimental Design. Representative figure showing experimental design and animal groups. HI, Hypoxia- Ischemia; IHC, immunohistochemistry; DHE, Dihydroethidium. (TIF 2430 kb) [file 12974_2019_1559_MOESM1_ESM.tif]

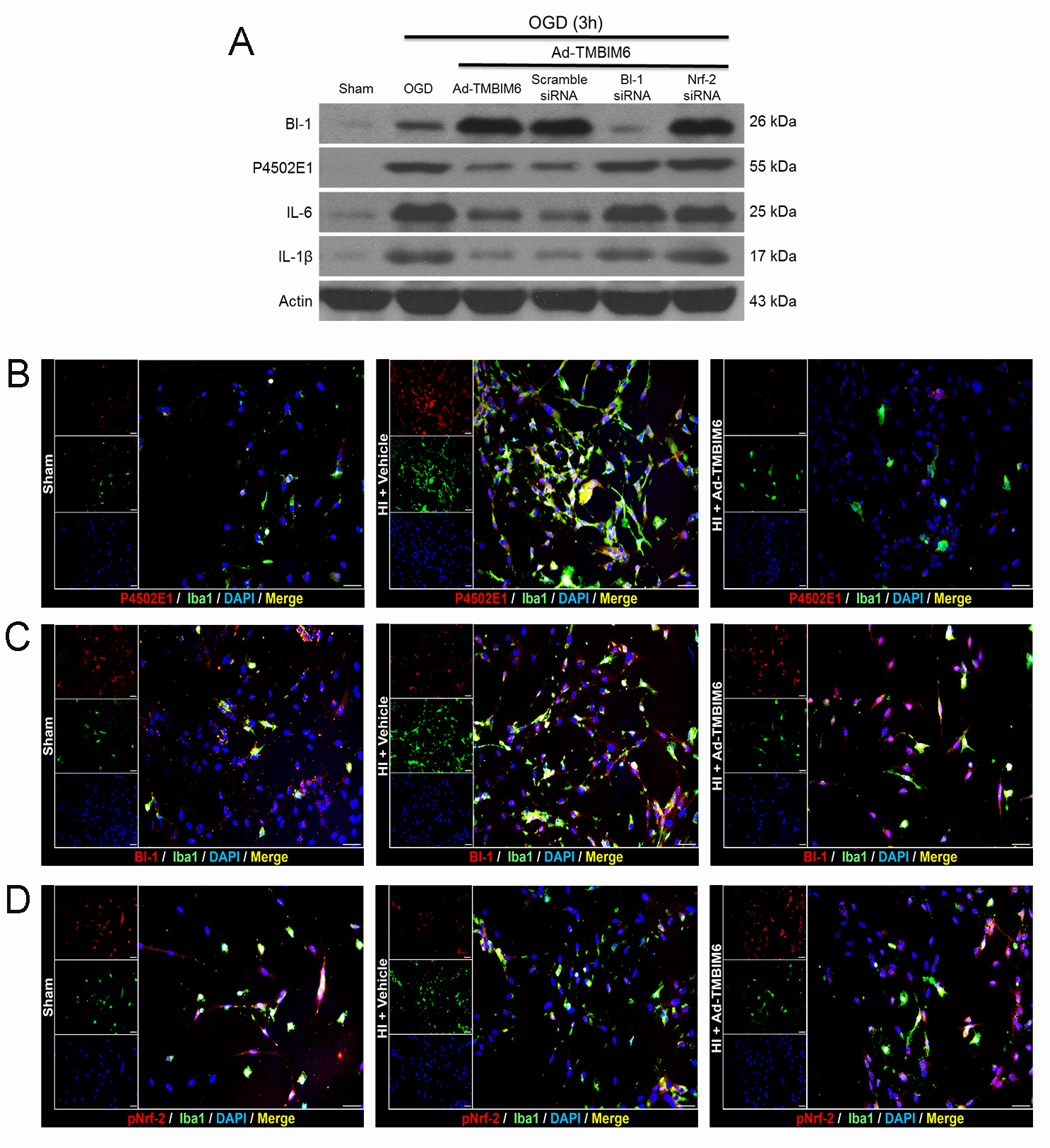

Supplement: Supplementary file 2 — Ad-TMBIM6 attenuates inflammation in primary microglial cells after OGD. Representative western blot bands of BI-1, P4502E1, IL-6 and IL-1β (A). Immunofluorescent staining of Iba-1 with P4502E1, BI-1 or pNrf-2 (B-D). (Green was for microglial staining, Red was for P4502E1, BI-1 or pNrf-2. Merge showed the co-localization of P4502E1, BI-1 or pNrf-2 on microglia. Scale bar 50 μm). (TIF 3861 kb) [file 12974_2019_1559_MOESM2_ESM.tif]

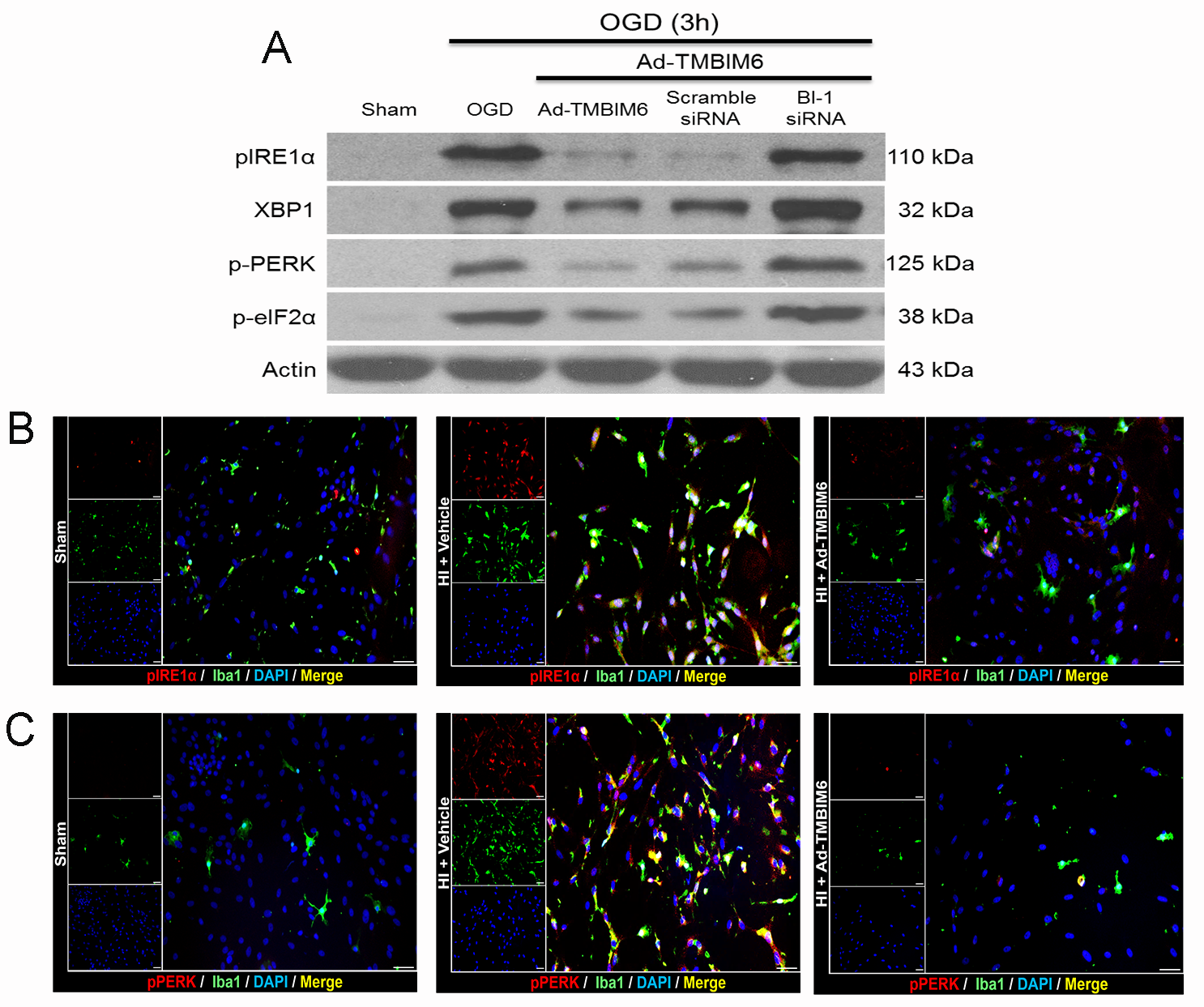

Supplement: Supplementary file 3 — Ad-TMBIM6 attenuates ER stress receptors’ signaling in primary microglial cells after OGD. Representative western blot bands of pIRE1α, XBP1, pPERK and peIF2α (A). Immunofluorescent staining of Iba-1 with pIRE1α or pPERK (B-C). (Green was for microglial staining, Red was for pIRE1α or pPERK. Merge showed the co-localization of pIRE1α or pPERK on microglia. Scale bar 50 μm). (TIF 2942 kb) [file 12974_2019_1559_MOESM3_ESM.tif]
